# Controllable Growth of Monolayer and Bilayer WSe_2_ by Liquid-Phase Precursor via Chemical Vapor Deposition for Photodetection

**DOI:** 10.3390/nano14242021

**Published:** 2024-12-16

**Authors:** Siyuan Wang, Pinyi Wang, Hailun Tang, Shilong Yu, Huihui Ye, Xinyu Fang, Jing Ding, Yang Yang, Hai Li

**Affiliations:** School of Flexible Electronics (Future Technologies), Institute of Advanced Materials (IAM), Nanjing Tech University (NanjingTech), 30 South Puzhu Road, Nanjing 211816, China

**Keywords:** monolayer WSe_2_, bilayer WSe_2_, liquid precursor, optoelectronic performance, chemical vapor deposition

## Abstract

Two-dimensional WSe_2_ nanosheets have received increasing attention due to their excellent optoelectronic properties. Solid precursors, such as WO_3_ and Se powders, have been extensively employed to grow WSe_2_ nanosheets by the chemical vapor deposition (CVD) method. However, the high melting point of WO_3_ results in heterogeneous nucleation sites and nonuniform growth of the WSe_2_ nanosheet. By dissolving WO_3_ powder in a NaOH solution, we report a facile and uniform growth of monolayer and bilayer WSe_2_ nanosheets on a SiO_2_/Si substrate at a large scale using liquid precursor by the CVD method. The size and thickness of the WSe_2_ nanosheets were controlled by modulating the precursor concentration and growth temperature. The as-prepared monolayer and bilayer WSe_2_ nanosheets were well characterized by optical microscopy, atomic force microscopy, and Raman and photoluminescence spectroscopy. With the increase in precursor concentration, the size of the monolayer WSe_2_ increased up to 120 μm. Bilayer WSe_2_ nanosheets were grown at higher temperatures. The photosensitivity of the bilayer WSe_2_ was one order of magnitude higher than that of the monolayer WSe_2_. The carrier mobility, specific detectivity, photoresponsivity, and external quantum efficiency of the bilayer WSe_2_ were about two orders of magnitude higher than those of the monolayer WSe_2_. Our method opens up a new avenue to grow monolayer and bilayer WSe_2_ for optoelectronic applications.

## 1. Introduction

As an important two-dimensional (2D) semiconductor material, WSe_2_ has gained tremendous attention due to its unique optical and electrical properties. Similar to other transition metal dichalcogenides (TMDCs; e.g., MoS_2_, WS_2_, and MoSe_2_), WSe_2_ also shows a thickness-dependent band gap as well as excellent optoelectronic performance. The band structure of WSe_2_ exhibits an indirect-to-direct transition as the thickness decreases from bilayer (2L) to monolayer (1L) [1,2], which greatly enhances the photoelectronic conversion efficiency. The monolayer WSe_2_ also exhibits quite high photoluminescence (PL) and high photo gain and specific detectivity [3]. While different from other TMDCs, WSe_2_ exhibits promising ambipolar behavior by modulating the interface and metal contact electrode [4,5,6,7,8]. In addition, a self-powered photovoltaic photodetector with fast photoresponse can be achieved by contacting the WSe_2_ nanosheet with TiN/Ti metal electrodes [9]. Interestingly, the WSe_2_ nanosheet also shows the lowest thermal conductivity among the layered TMDC materials [10]. It also has extensive applications in hydrogen evolution reactions [11], light-emitting diodes [12], photodetectors [13], and field-effect transistors [14]. To date, researchers have developed various methods to prepare WSe_2_ nanosheets, including mechanical exfoliation (ME) [1], physical vapor deposition (PVD) [15,16], chemical vapor deposition (CVD) [5,7,17,18,19,20], and liquid exfoliation [21,22,23]. Among them, CVD is a widely used method because of its advantages of low cost, high-quality growth, good uniformity, and high controllability of thickness [24,25,26,27].

It is well known that the successful CVD growth of WSe_2_ nanosheets is highly dependent on the type of transition metal and selenium precursors. Solid precursors such as transition metal oxides (e.g., WO_3_) and selenium powders have been extensively employed to grow WSe_2_ nanosheets on a substrate during the CVD process. However, the high melting point of WO_3_ powder (~1475 °C) hinders its uniform vapor phase distribution in the tube furnace [28,29,30], leading to nonuniform nucleation sites on the substrate. Therefore, heterogeneous WSe_2_ nanosheets with mixed layer numbers can be obtained. Various solutions have been proposed to grow uniform WSe_2_ nanosheets on substrates in large sizes, such as introducing halide salts to reduce the melting point of WO_3_ [31,32], adding Te powder as a growth promoter [33], and optimizing the growth temperature as well as the distance between substrate and precursor [28,29]. However, it is still a challenge to grow large WSe_2_ nanosheets with homogeneous morphology, large domain size, and low density of defects by using a solid precursor, which, in turn, influences the electrical and optoelectronic properties of the as-grown WSe_2_ nanosheets.

Recently, a liquid-phase precursor instead of solid powder has been developed to grow TMDC nanosheets uniformly at wafer scale [34,35,36,37]. Compared to with the solid precursor, the precursor concentration can be well controlled by dissolving the precursor salt in a certain solvent, which is suitable for making the mixture of different salts grow a heterostructure nanosheet. In addition, the liquid precursor can be uniformly distributed on the substrate by the spin coating method. Thus, the nucleation density can be precisely modulated by depositing a tiny amount of precursor solution on a substrate. As a consequence, monolayer TMDC nanosheets can be grown effectively by avoiding the supersaturation state of the precursor vapor in the solid precursor method [38,39]. Moreover, the precursor salt can be easily converted into the molten state due to its lower melting point (650–850 °C). Uniform and high-quality TMDC nanosheets, such as monolayer MoS_2_ and WS_2_ as well as hetero-bilayer MoS_2_/WS_2_, have been successfully grown by using the liquid precursor method [36,40]. However, the growth of 1L and 2L WSe_2_ nanosheets using the liquid precursor remains poorly explored. Therefore, it is desirable to develop a liquid-phase precursor to achieve controllable growth of 1L and 2L WSe_2_.

Herein, we report the uniform growth of 1L and 2L WSe_2_ nanosheets on a SiO_2_/Si substrate in at a large scale with the CVD method using a liquid-phase precursor. The liquid precursor is firstly obtained by dissolving WO_3_ powder in NaOH solution and then depositing it on the SiO_2_/Si substrate with a controlled concentration. By optimizing the precursor concentration and growth temperature, the size and thickness of the WSe_2_ nanosheets were well regulated. The as-prepared 1L and 2L WSe_2_ nanosheets were characterized by optical microscopy (OM), atomic force microscopy (AFM), and Raman and PL spectroscopy. Compared with 1L WSe_2_, the 2L WSe_2_ shows one order of magnitude improvement in terms of photosensitivity. In addition, the photoresponsivity, external quantum efficiency (EQE), carrier mobility, and specific detectivity of 2L WSe_2_ are two orders of magnitude higher than those of 1L WSe_2_, indicating that the 2L WSe_2_ could be promising candidate for optoelectronic devices.

## 2. Materials and Methods

### 2.1. Growth of Monolayer and Bilayer WSe_2_ Nanosheets by Chemical Vapor Deposition

The CVD process was performed in a single-heating-zone tube furnace under ambient pressure. In order to dissolve the WO_3_ powder (99.9%, Aladdin, Riverside, CA., USA) completely, 0.2 g of WO_3_ powder was added to NaOH solution (10 mL, 10 mg/mL) in water bath at 80 °C to form WO_3_ precursor solution with concentration of 20 mg/mL. The as-prepared WO_3_ precursor solution was diluted to 5, 10, and 15 mg/mL, respectively. The 300 nm SiO_2_/Si substrate was first immersed in a mixture of concentrated sulfuric acid and hydrogen peroxide at 120 °C for 30 min, and then washed by deionized water and dried by N_2_. The as-cleaned SiO_2_/Si substrate was soaked in a mixture of WO_3_ precursor solution and sodium cholate (1 mg/mL, 98%, Aladdin) with a volume ratio of 5:1 for 1 min. The sodium cholate was used as promoter to accelerate the growth of WSe_2_ [38,41,42]. Due to the hydrophilicity of the SiO_2_/Si substrate, the precursor was uniformly deposited on the substrate. The precursor-coated SiO_2_/Si substrate was slowly dried by N₂ and then put face-down on a quartz boat in the center of a tube furnace. Another quartz boat with 0.15 g Se powder (99%, Alfa Aesar, Ward Hill, MA, USA) was placed upstream of the tube. For the growth of 1L WSe_2_ nanosheet, the temperature was controlled at 800 °C, and the growth time was kept for 5 min with 20 sccm N_2_ and 10 sccm H_2_ used as carrier gas. The growth of 2L WSe_2_ nanosheet was the same as that of 1L WSe_2_ nanosheet, except the temperature was controlled at 900 °C.

### 2.2. Characterizations

An optical microscope (ECLIPSE LV100ND, Nikon, Tokyo, Japan) was used for a preliminary check on the location, shape, and thickness of the as-grown samples. An atomic force microscope (Dimension ICON with Nanoscope V controller, Bruker, Billerica, MA, USA) was used to characterize the thickness of 1L and 2L WSe_2_ nanosheets. The Raman and photoluminescence (PL) spectra of 1L and 2L WSe_2_ nanosheets were collected on a LabRAM HR Evolution Raman spectrometer (Horiba Jobin Yvon, Longjumeau, France) with a 532 nm laser focused through a 100 × objective lens at room temperature. The electrical performance was measured by a Keithley 4200 semiconductor device parameter analyzer (Keithley 4200-SCS, Tektronix, Beaverton, OR, USA).

### 2.3. Device Fabrication and Photodetection

A 300-mesh TEM mesh was used as mask in thermal evaporator. Then, 3 nm thick chromium and 50 nm thick gold films were deposited on 1L and 2L WSe_2_ nanosheets to obtain the device for measuring optoelectronic and electrical performance. The Keithley 4200 semiconductor characterization system was used to monitor the real-time current change in the WSe_2_ devices. The optoelectronic performance test was recorded under blue (405 nm), green (532 nm), and red (633 nm) lasers.

## 3. Results

**Scheme 1:**
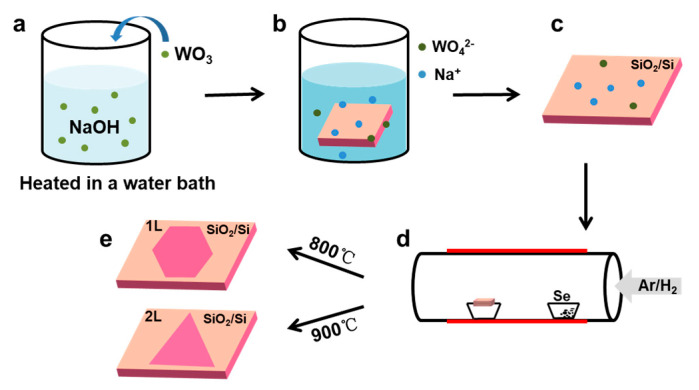
Schematic illustration of the CVD synthesis of WSe_2_ nanosheets using a liquid-phase precursor. (**a**) Preparation of the liquid-phase Na_2_WO_4_ precursor by dissolving WO_3_ powder in a NaOH solution. (**b**) Soaking the SiO_2_/Si substrate in a Na_2_WO_4_ solution for 1 min. (**c**) The precursor was uniformly distributed on the SiO_2_/Si substrate. (**d**) The growth of the WSe_2_ nanosheet by CVD method with Ar/H_2_ as carrier gas. (**e**) The growth of 1L and 2L WSe_2_ nanosheets by CVD at 800 and 900 °C, respectively.

The growth of the WSe_2_ nanosheet is shown in Scheme 1. Firstly, WO_3_ powder was dissolved in NaOH solution at a certain concentration. In order to accelerate the dissolution of WO_3_, the NaOH solution was then immersed into a water bath at 80 °C (Scheme 1a). The dissolution of WO_3_ in the NaOH solution can be described by the following reaction.

2NaOH + WO_3_ → Na_2_WO_4_ + H_2_O (1)

Thus, a Na_2_WO_4_ solution was obtained after WO_3_ was completely reacted with NaOH. A freshly cleaned SiO_2_/Si substrate was then immersed into the Na_2_WO_4_ solution for some time to obtain a Na_2_WO_4_-coated substrate (Scheme 1b,c). The CVD process was performed by putting Se powder upstream of the Na_2_WO_4_-coated SiO_2_/Si substrate in a single-heating-zone tube furnace with Ar/H_2_ as the carrier gas (Scheme 1d). By controlling the growth temperature, 1L and 2L WSe_2_ nanosheets were grown at 800 and 900 °C (Scheme 1e), respectively. The growth of a WSe_2_ nanosheet by CVD can be described as the following chemical reaction.

Na_2_WO_4_ + 3Se → WSe_2_ + Na_2_Se + 2SeO_2_ (2)

**Figure 1:**
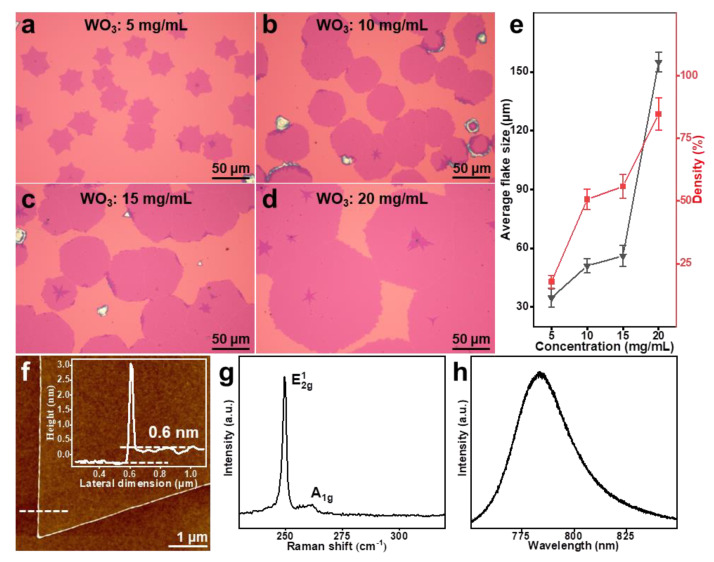
OM images of CVD-grown 1L WSe_2_ nanosheets by dissolving WO_3_ powder in NaOH solution at concentrations of (**a**) 5, (**b**) 10, (**c**) 15, and (**d**) 20 mg/mL, respectively. (**e**) The average flake size and density of 1L WSe_2_ as a function of WO_3_ concentration. (**f**) AFM image of the as-grown 1L WSe_2_ nanosheet. Inset shows the height profile of the 1L WSe_2_ nanosheet across the white dashed line. (**g**) Raman and (**h**) PL spectra of the 1L WSe_2_ nanosheet.

At first, the influence of WO_3_ concentration on the growth of the WSe_2_ nanosheet was investigated. Figure 1a–d show the optical microscopy (OM) images of 1L WSe_2_ nanosheets grown at 800 °C by dissolving WO_3_ in NaOH solutions with concentrations of 5, 10, 15, and 20 mg/mL, respectively. With the increased concentration of WO_3_, the size and density of the WSe_2_ nanosheets also increased quickly. When WO_3_ with concentration of 5 mg/mL was used as precursor, symmetric six-pointed WSe_2_ stars with sizes of 34.7 ± 4.7 μm were obtained (Figure 1a). As the concentration of WO_3_ increased to 10 mg/mL, the as-grown WSe_2_ nanosheets showed hexagonal shapes with sizes of 51.0 ± 3.6 μm (Figure 1b). The size of the WSe_2_ nanosheets gradually increased to 56.0 ± 5.3 and 155.0 ± 5.0 μm as the concentration of WO_3_ increased to 15 and 20 mg/mL, respectively (Figure 1c,d). Meanwhile, the densities of the WSe_2_ nanosheets also increased with the increasing concentrations of WO_3_. In order to measure the percentage of the surface area of the SiO_2_/Si substrate covered by WSe_2_ nanosheets, ImageJ software was used for statistical measurement (Figure S1 in Supporting Information (SI)). When 5 mg/mL of WO_3_ was used as the precursor, only 17.9 ± 2.6% area of the SiO_2_/Si substrate was covered by WSe_2_ nanosheets. The density of the WSe_2_ nanosheets further increased to 50.7 ± 4.0% as 10 mg/mL WO_3_ was used. It slightly increased to 55.8 ± 4.8% when the concentration of WO_3_ increased to 15 mg/mL, while the density of the WSe_2_ nanosheets largely increased to 84.7 ± 6.4% as the concentration of WO_3_ increased to 20 mg/mL. These results indicated that the size and density of WSe_2_ nanosheets are highly dependent on the concentration of WO_3_ dissolved in the NaOH solution, as indicated in Figure 1e. At a low concentration of WO_3_ solution (5 mg/mL), the nucleation density is also low, and the as-grown WSe_2_ nanosheets are small and sparsely distributed on the substrate. With the increasing concentration of WO_3_ (10 and 15 mg/mL), the amount of Na_2_WO_4_ is also increased, which results in the increased size of the WSe_2_ nanosheets. A higher concentration of WO_3_ solution (20 mg/mL) can provide a sufficient amount of Na_2_WO_4_ precursor for growing larger WSe_2_ nanosheets. In addition to the concentration of WO_3_, the distance between the Se powder and Na_2_WO_4_-coated SiO_2_/Si substrate also affects the growth of the WSe_2_ nanosheet (Figure S2 in SI). As shown in Figure S2a in SI, the precursor-coated SiO_2_/Si substrate is placed at the center of the tube furnace, where the temperature is the highest. The Se powder in the ceramic boat is placed at the left end of the furnace, a certain distance away from the SiO_2_/Si substrate. Since there is a temperature gradient in the tube furnace, the temperature of the Se boat decreases with the increasing distance. Therefore, the Se powder is evaporated quickly when the distance is 15.5 cm, resulting in oversaturation of Se vapor near the SiO_2_/Si substrate, while insufficient Se vapor is provided for the growth of the WSe_2_ nanosheet when the distance is set to 17 cm because of the lower temperature. As shown in Figure S2b in SI, large monolayer WSe_2_ nanosheets and thick flakes are grown when the distance is 15.5 cm, while very small monolayer WSe_2_ nanosheets are grown at a distance of 17 cm (Figure S2e in SI), confirming the important influence of distance on the growth of WSe_2_ nanosheets. The size of the monolayer WSe_2_ nanosheet increases as the distance decreases from 17 cm to 16.5 cm (Figure S2d in SI). As shown in Figure S2c in SI, the large monolayer WSe_2_ nanosheets with clean surfaces are grown when the distance is 16 cm, indicating it is the optimal distance.

Figure 1f–h shows the AFM image and Raman and PL spectra of a WSe_2_ nanosheet grown at 800 °C by using 10 mg/mL of WO_3_. The AFM image shown in Figure 1f indicates the smooth morphology of the WSe_2_ nanosheet. The measured thickness of the WSe_2_ nanosheet is 0.6 nm, indicating the monolayer structure. Raman and PL spectroscopy were then used to confirm the thickness and crystalline quality of the WSe_2_ nanosheets shown in Figure 1a–d. There are two representative Raman peaks located at ~250 cm^−1^ ($E_{2g}^{1}$) and ~260 cm^−1^ ($A_{1g}$) (Figure 1g), indicating the good crystalline structure of the WSe_2_ nanosheets. The absence of the $B_{2g}^{1}$ peak at ~308 cm^−1^ suggests the monolayer structure of the as-grown WSe_2_ nanosheets. Moreover, a symmetric PL peak located at 783.1 nm further confirms the existence of 1L WSe_2_ (Figure 1h) [1,5].

**Figure 2:**
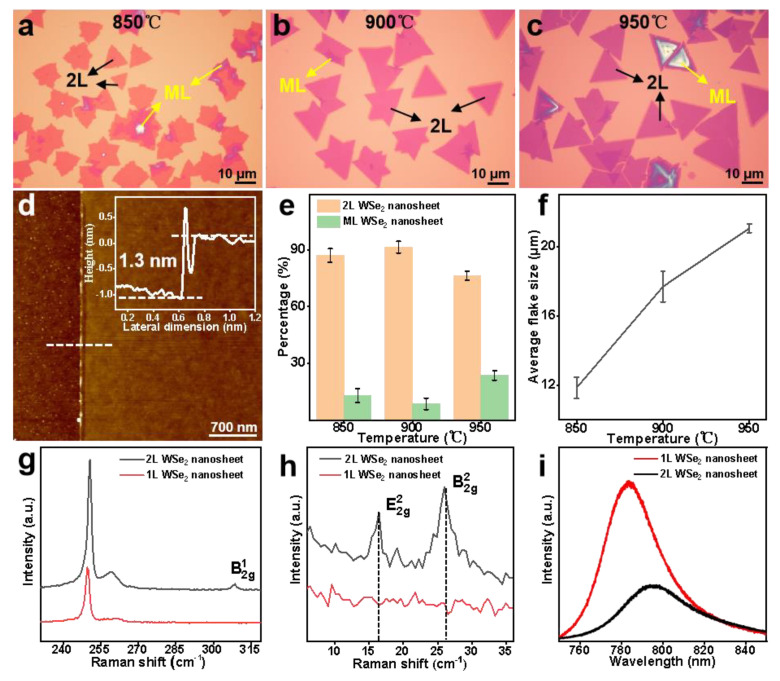
OM images of CVD-grown 2L WSe_2_ nanosheets at (**a**) 850, (**b**) 900, and (**c**) 950 °C, respectively. (**d**) AFM image of the as-grown 2L WSe_2_ nanosheet. Inset shows the height profile of the 2L WSe_2_ nanosheet across the white dashed line. (**e**) The statistical histogram of 2L and ML WSe_2_ nanosheets grown at different temperatures. (**f**) The average flake size of 2L WSe_2_ as a function of growth temperature. (**g**) High-frequency Raman, (**h**) low-frequency Raman, and (**i**) PL spectra of 1L and 2L WSe_2_ nanosheets.

We found that the growth temperature also has an important effect on the growth of WSe_2_ in addition to the precursor concentration and the distance between the substrate and Se powder. Figure 2a–c show OM images of WSe_2_ nanosheets grown at 850, 900, and 950 °C, respectively. When the growth temperature was set to 850 °C, many WSe_2_ nanosheets with darker contrast than the 1L WSe_2_ nanosheet were observed (Figure 2a). AFM was used to measure the thickness of these WSe_2_ nanosheets. As shown in Figure 2d, the thickness of the WSe_2_ nanosheet was measured to be 1.3 nm, as expected for the 2L nanosheet [1]. Moreover, there are also some multilayer (ML) WSe_2_ nanosheets shown in Figure 2a, as indicated by the yellow arrows. The size of the 2L and ML WSe_2_ nanosheets grown at 850 °C was usually in the range of 10 to 20 μm. The number of 2L and ML WSe_2_ nanosheets was then counted. It was found that more than 87.0 ± 3.6% of the WSe_2_ nanosheets were 2L, as shown in Figure 2e. As the growth temperature increased to 900 °C, uniform 2L WSe_2_ nanosheets with sizes larger than 20 μm were grown, and the surfaces of the WSe_2_ nanosheets were cleaner than that of the WSe_2_ nanosheet grown at 850 °C. In this case, the percentage of 2L WSe_2_ nanosheets increased to 91.3 ± 3.1% (Figure 2e) while the percentage of 2L WSe_2_ nanosheets decreased to 76.3 ± 2.5% as the temperature further increased to 950 °C. As indicated in Figure 2c, some ML WSe_2_ nanosheets were observed. With the increase of in temperature, the longitudinal growth of WSe_2_ nanosheets was promoted because of the increased concentration of selenium vapor, which accelerated the reaction with Na_2_WO_4_ [38,43]. As the temperature increased from 850 to 950 °C, the size of the 2L WSe_2_ nanosheets increased from 11.8 ± 0.6 to 21.2 ± 0.2 μm. Therefore, the optimal temperature for growing 2L WSe_2_ nanosheets was set to 900 °C, which is higher than that of the 1L nanosheets. The reasons can be explained as follows. It is well known that the temperature gradients within the tube furnace play an important role in controlling the growth of 2D TMDC nanosheets in the CVD process. In our work, the temperature gradients also influenced the growth of the WSe_2_ nanosheets. As shown in Figure S3 in SI, the Se powders were placed in a boat, which was just out of the left end of the furnace. When the temperature of the furnace center was set to 800 °C, the temperature of this boat might be a bit higher than the melting point of Se, leading to slow evaporation of the Se powder. Therefore, a low concentration of Se vapor could be transported to the Na_2_WO_4_-coated SiO_2_/Si substrate for growing large 1L WSe_2_ nanosheets. As the temperature of the furnace center increased to 900 °C, the temperature of the Se boat further increased. Thus, a higher concentration of Se vapor could be transported to the Na_2_WO_4_-coated SiO_2_/Si substrate, which accelerated the vertical growth of the WSe_2_ nanosheet while the lateral growth was a bit decelerated. In this case, 2L WSe_2_ nanosheets with smaller sizes than the 1L WSe_2_ nanosheets were obtained. We also investigated the influence of growth time on the formation of 1L and 2L WSe_2_ nanosheets. Figure S4 in SI shows the 1L and 2L WSe_2_ nanosheets grown under the same conditions with growth times of 1, 5, and 10 min, respectively. The growth time shows a slight influence on the size of the 1L WSe_2_ nanosheets. As shown in Figure S4a,b in SI, the size of the 1L WSe_2_ nanosheets slightly increased as the growth time was changed from 1 min to 5 min. However, there were small, irregular WSe_2_ flakes grown on top of the 1L WSe_2_ as the growth time increased to 10 min. Figure S4d–f in SI show the OM images of 2L WSe_2_ nanosheets at different growth times. The size of 2L WSe_2_ showed little change at different growth times.

It is worth noting that the 2L WSe_2_ nanosheets are triangle shaped, which are different from the hexagonal shape of the 1L WSe_2_ nanosheets. This could be explained as follows. The 1L WSe_2_ nanosheets grown at lower temperature and precursor concentration exhibit symmetric six-pointed star shapes, which could be assigned to the slower growth rate and the surface diffusion energy (Figure 1a) [31]. When the concentration of WO_3_ increases from 5 to 10 mg/mL, the ratio of W to the Se precursor increases, which promotes the lateral growth of the WSe_2_ nanosheets. Thus, the 1L WSe_2_ nanosheets are changed into hexagonal shapes. With the increase in temperature (~900 °C), the concentration of Se vapor increases, accelerating the vertical growth to obtain 2L WSe_2_ nanosheets [43,44]. On the other hand, the kinetic-controlled attachment and detachment of atoms also affect the domain morphology. When 1L WSe_2_ nanosheets are grown at a low precursor concentration under a relatively low temperature (800 °C), the growth rates at the W-terminated and Se-terminated ends are almost equal due to the low nucleation density, resulting in a hexagonal domain with zigzag edges. With the increase in the W precursor concentration, the hexagonal-shaped domain grows continuously, which is energy favorable. When the 2L WSe_2_ nanosheets are grown at a higher temperature (900 °C), due to the increased concentration of Se vapor, the growth rate of the Se-terminated end is faster than that of the W-terminated end. In this case, the domain would be changed from a hexagon to a triangle. Moreover, the reaction rate is quite fast at high temperatures, and the W and Se atoms are not easy to diffuse to the energy-favorable position, resulting in the size of the 2LWSe_2_ nanosheet being smaller than that of the 1L WSe_2_ nanosheet [44]. Such a kind of shape evolution was also reported in the CVD growth of MoS_2_ nanosheets [45].

Figure 2g–i show the ultralow frequency (ULF) and high-frequency Raman and PL spectra of the 2L WSe_2_ nanosheet. There is a $B_{2g}^{1}$ peak at ∼309 cm^−1^ in the high-frequency Raman spectrum of the WSe_2_ nanosheets (Figure 2g) that is absent in the 1L WSe_2_ nanosheet, implying they are of 2L or larger thickness. As shown in Figure 2h, the WSe_2_ nanosheet shows a shear mode peak at 16.4 cm^−1^ and a layer breathing mode peak at 26.1 cm^−1^ in the ULF Raman spectrum, which are the characteristic ULF peaks of 2L WSe_2_ [46], confirming the 2L thickness of as-grown WSe_2_ nanosheets. As shown in Figure 2i, the 2L WSe_2_ shows a relatively weak PL peak at 794.4 nm, while the 1L WSe_2_ shows a strong peak at 783.1 nm in the PL spectra, which are consistent with previous reports [19,47].

**Figure 3:**
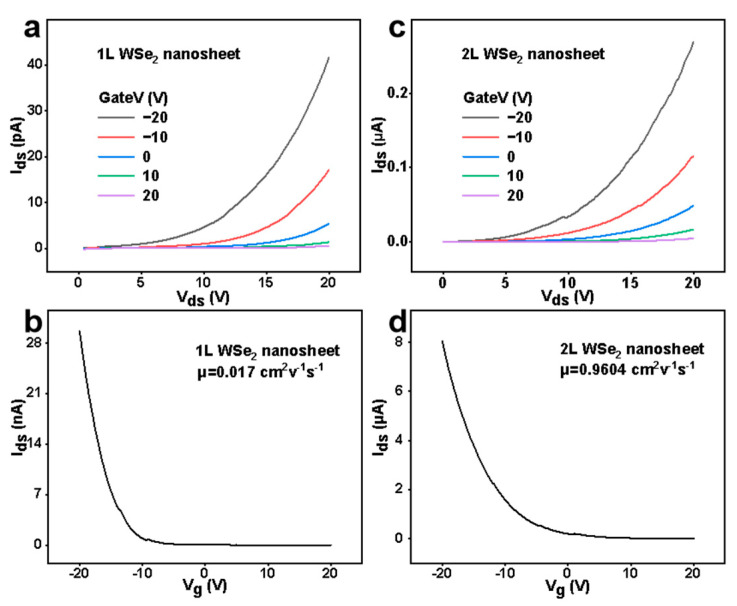
Output and transfer curves of devices based on (**a**,**b**) 1L and (**c**,**d**) 2L WSe_2_ nanosheets.

In order to evaluate the optoelectronic properties of 1L and 2L WSe_2_ nanosheets grown on SiO_2_/Si substrate, back-gate field-effect transistor devices were fabricated by using a TEM grid as a mask in the thermal evaporator. The typical I_ds_-V_ds_ characteristics of 1L and 2L WSe_2_ devices are shown in Figure 3a,c. The drain current decreases with the increase in the gate voltage, indicating the p-type behavior of the as-fabricated WSe_2_ devices. The I_ds_-V_g_ curves of the 1L and 2L WSe_2_ devices are plotted in Figure 3b,d. The I_ds_ decreases monotonically with the increase in *V_g_*, also confirming the p-type behavior. The field-effect mobility of WSe_2_ devices is calculated by using the following formula.

(3) $$\mu =\frac{L}{W\times \left(\frac{\varepsilon_{0}\varepsilon_{r}}{d}\right){\times V}_{ds}}\frac{dI_{ds}}{dV_{g}}$$

where *L* and *W* are the length and width of the device channel, $\varepsilon_{0}$ is 8.854 × 10^−12^ Fm^−1^, $\varepsilon_{r}$ for SiO_2_ is 3.9, and *d* is the thickness of SiO_2_ (300 nm). The $dI_{ds}/dV_{g}$ represents the slope extracted from the linear region of the transfer curve [48,49].

The calculated mobilities were ~0.017 and ~0.9604 cm^2^V^−1^s^−1^ for 1L and 2L WSe_2_ nanosheets when a constant voltage of 10V was applied between the source and drain, respectively, which are comparable to previous reports [3,50]. The 2L WSe_2_ nanosheet showed much higher mobility than that ofdid the 1L WSe_2_ nanosheet, which is consistent with previous reports [17,47].

**Figure 4:**
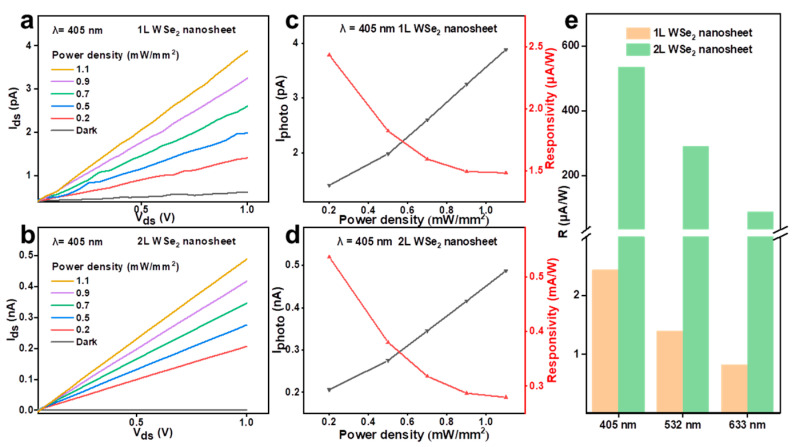
The output characteristics of photodetectors based on (**a**) 1L and (**b**) 2L WSe_2_ nanosheets at Vg = 0 V illuminated by 405 nm laser with various power densities. The photocurrent and photoresponsivity of (**c**) 1L and (**d**) 2L WSe_2_ devices illuminated by 405 nm lasers as a function of laser power density. (**e**) The statistical histogram of the photoresponsivity of photodetectors based on 1L and 2L WSe_2_ nanosheets.

Following the electrical measurement, the optoelectronic performance of the 1L and 2L WSe_2_ devices was investigated under 405, 532, and 633 nm lasers with different power densities, respectively. As shown in Figure 4a, the I_ds_ of the 1L WSe_2_ device linearly increased to 0.6 pA as the V_ds_ increased from 0 to 1.0 V under dark conditions. When the 1L WSe_2_ device was illuminated by a 405 nm laser with a power density of 0.2 mW/mm^2^, the I_ds_ increased to 1.4 pA as the V_ds_ increased from 0 to 1.0 V. As the power density of the 405 nm laser increased from 0.5 to 1.1 mW/mm^2^, the I_ds_ of the 1L WSe_2_ device also increased from 2.0 pA to 4.0 pA, indicating it has good optoelectronic performance under illumination. The I_ds_ of the 2L WSe_2_ device linearly increased to 0.3 pA as the V_ds_ increased from 0 to 1.0 V under dark conditions, as shown in Figure 4b. Surprisingly, the I_ds_ of the 2L WSe_2_ device greatly increased to 206 pA as the V_ds_ increased from 0 to 1.0 V when the device was illuminated by a 405 nm laser with a power density of 0.2 mW/mm^2^. The I_ds_ continuously increased under the 405 nm laser with increased power density. Similar phenomena were also observed in both 1L and 2L WSe_2_ devices under illumination with 532 and 633 nm lasers (Figure S5 in SI), indicating the I_ds_ has a strong dependence on the laser power density [51]. We also investigated the photoresponsivity (R), EQE, and specific detectivity (D*) of the 1L and 2L WSe_2_ devices by the following equations.

(4) $$R=I_{ph}/PS$$

where *I_ph_* is the photocurrent, *P* is the incident power density, and *S* is the effective illumination area of the device.

(5) EQE=hcR/eλ

where *h* is the Planck’s constant, *c* is the speed of light, *e* is the charge, and *λ* is the laser wavelength.

(6) $$D^{*}=\frac{R_{\lambda }S^{1/2}}{({2eI_{dark})}^{1/2}}$$

where *R_λ_* is responsivity, *S* is the effective irradiated area, *e* is the charge, and *I_dark_* is the dark current.

Figure 4c,d show the photoresponsivity and photocurrent characteristics of 1L and 2L WSe_2_ devices under the 405 nm laser. The photocurrent of the 1L and 2L WSe_2_ devices monotonously increased as the laser power density increased from 0.2 to 1.1 mW/mm^2^. The photoresponsivity of the 2L WSe_2_ photodetector was two orders of magnitude higher than that of the 1L WSe_2_ photodetector. Figure S6 in SI shows the plots of the photocurrent and photoresponsivity of the 1L and 2L WSe_2_ photodetectors as a function of laser power density under the 532 and 633 nm lasers, respectively, which are similar to the characteristics shown in Figure 4c,d. The 2L WSe_2_ nanosheets also showed much higher D* and EQE than those of the 1L WSe_2_ nanosheets under 405, 532, and 633 nm lasers. Table S1 in the SI compares the performance of this work with the previously reported photodetectors based on TMDC [52,53,54,55]. Compared with other photodetectors, our WSe_2_ devices show comparable performance. The aforementioned results show that the 2L WSe_2_ nanosheets exhibited much better performance than the 1L WSe_2_ nanosheets, implying the influence of thickness on the optoelectronic performance [19,20,56].

In addition to photoresponsivity, photosensitivity is also an important parameter to evaluate the performance of photodetectors [51,57,58]. Photosensitivity is usually described as the photocurrent-to-dark-current ratio (PDR), which is defined by the following equation.

(7) $$PDR=I_{photo}/I_{dark}$$

where *I_photo_* is the photocurrent under illumination, and *I_dark_* is the baseline current without illumination.

**Figure 5:**
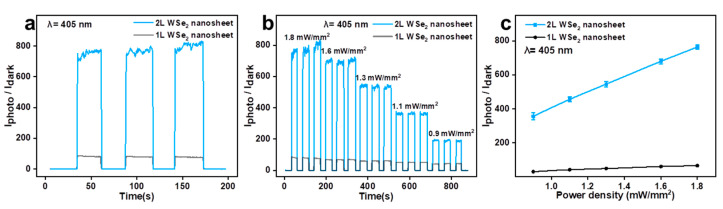
(**a**) The PDR plot of photodetectors based on 1L and 2L WSe_2_ nanosheets under a 405 nm laser with a power density of 1.8 mW/mm^2^. (**b**,**c**) The PDR plots of photodetectors based on the 1L and 2L WSe_2_ nanosheets under a 405 nm laser as a function of the laser power density.

Figure 5a shows the photosensitivity of 1L and 2L WSe_2_ devices as a function of time under illumination with a 405 nm laser with a power density of 1.8 mW/mm^2^ and under dark conditions. The photodetectors based on the 1L and 2L WSe_2_ nanosheets exhibited quite stable characteristics after multiple illumination–dark cycles. The PDR of the 1L WSe_2_ device was ~100, while the 2L WSe_2_ photodetector showed a PDR of ∼760 (blue curve in Figure 5a), which is about one 1 order of magnitude higher than that of the 1L WSe_2_ photodetector (black curve in Figure 5a). Similarly, the 2L WSe_2_ photodetector also shows a much higher PDR than that of the 1L WSe_2_ photodetector under 532 and 633 nm lasers (Figure S7 in SI), implying the 2L WSe_2_ nanosheet presents high photosensitivity and better optoelectronic performance. As shown in Figure 5b, the PDRs of the 1L and 2L WSe_2_ devices under the 405 nm laser were plotted as a function of laser power density. With the decrease in laser power density, the PDR decreased accordingly, indicating it is highly dependent on the laser power density [58]. As shown in Figure 5c, compared with the 1L WSe_2_ photodetector, the PDR of the 2L WSe_2_ photodetector greatly increased with the increasing laser power density under illumination with a 405 nm laser. Moreover, the PDRs of the 2L WSe_2_ photodetectors also showed one order of magnitude higher than those of the 1L WSe_2_ photodetectors under illumination of 532 and 633 nm lasers with various power densities (Figure S8 in SI), respectively. Therefore, the photodetector based on the 2L WSe_2_ nanosheet showed much better photodetection performance than that based on the 1L WSe_2_ nanosheet.

In addition, the photoresponse time and recovery time of the 1L WSe_2_ photodetector are 1.5 s and 1.5 s (Figure S9 in SI), respectively, while the photoresponse time and recovery time of the 2L WSe_2_ photodetector are 0.6 s and 0.5 s, respectively, indicating the 2L WSe_2_ photodetector shows a much faster response than the 1L WSe_2_ photodetector under illumination with a 405 nm laser. Similar phenomena were also observed for 1L and 2L WSe_2_ photodetectors illuminated by 532 and 633 nm lasers (Figure S9 in SI). The detailed optoelectronic performance of the 1L and 2L WSe_2_ devices is listed in Table 1. Compared with the 1L WSe_2_ photodetector, the 2L WSe_2_ photodetector shows much better performance.

## 4. Conclusions

In summary, 1L and 2L WSe_2_ nanosheets were successfully grown on a SiO_2_/Si substrate by using liquid precursor-mediated CVD. Different from the solid precursor used in typical CVD, WO_3_ powder was dissolved in a NaOH solution and used as a liquid precursor. The size and thickness of the WSe_2_ nanosheets were well regulated by optimizing the precursor concentration and growth temperature. As the precursor concentration increased, the size of the 1L WSe_2_ nanosheet also increased. The 1L WSe_2_ nanosheets were grown at 800 °C, while 2L WSe_2_ nanosheets were obtained at 900 °C. The OM, AFM, and Raman and PL spectroscopy characterizations confirmed that the 1L and 2L WSe_2_ nanosheets were of good quality. Compared with the 1L WSe_2_ nanosheet, the device based on the 2L WSe_2_ nanosheet showed much better optoelectronic performance. The photoresponsivity, external quantum efficiency (EQE), carrier mobility, photoresponsivity, and specific detectivity of the 2L WSe_2_ nanosheet were about two orders of magnitude higher than those of the 1L WSe_2_ nanosheet. Moreover, the 2L WSe_2_ nanosheet also showed a PDR about ten times higher than 1L WSe_2_ nanosheet. Our results indicate that the 2L WSe_2_ nanosheet shows much better optoelectronic performance than the 1L WSe_2_ nanosheet and could be a promising candidate for high-performance optoelectronic devices.

## Figures and Tables

**Table 1:** The detailed photocurrent, photoresponsivity, specific detectivity, external quantum efficiency, PDR, response time, and mobility of 1L and 2L WSe_2_ devices under 405, 532, and 633 nm lasers, respectively.

Device	Wavelength	I_Photo_ (pA)	R (A/W)	D* (cm·Hz^1/2^W^−1^)	EQE (%)	PDR	T_r_ (s)	μ (cm^2^V^−1^S^−1^)
1L	405 nm	1.41	2.44 × 10^−6^	1.45 × 10^9^	7.46 × 10^−4^	66	1.5	0.017
	532 nm	1.14	1.40 × 10^−6^	8.20 × 10^8^	3.26 × 10^−4^	82	1.4	
	633 nm	1.10	8.30 × 10^−7^	5.24 × 10^8^	1.63 × 10^−4^	76	1.4	
2L	405 nm	206	5.36 × 10^−4^	4.42 × 10^11^	1.64 × 10^−1^	761	0.6	0.96
	532 nm	158	2.91 × 10^−4^	2.35 × 10^11^	6.80 × 10^−2^	860	0.7	
	633 nm	77.6	8.79 × 10^−5^	6.91 × 10^10^	1.72 × 10^−2^	790	0.7	

## Data Availability

Data can be available upon request from the authors.

## Supplementary Materials

The following supporting information can be downloaded at: https://www.mdpi.com/article/10.3390/nano14242021/s1, Figure S1: The statistics investigation of the surface area percentage of SiO_2_/Si substrate covered by WSe_2_ nanosheets. (a) Color image of WSe_2_ nanosheets on SiO_2_/Si substrate. (b) Gray-scale image of (a) processed by ImageJ (version 1.54g). (c) The coverage of WSe_2_ nanosheets on SiO_2_/Si substrate was statistically analyzed by ImageJ. Figure S2: (a) Schematic illustration of the evaporation of Se powder at various distances away from the SiO_2_/Si substrate placed at the center of a CVD tube furnace. (b–e) OM images of 1L WSe_2_ nanosheets grown at distances between the substrate and selenium powder of (b) 15.5, (c) 16.0, (d) 16.5, and (e) 17.0 cm, respectively. (f) The average flake size and density of 1L WSe_2_ nanosheets as a function of distance. Figure S3: Schematic illustration of the evaporation of Se powder at (a) 800 and (b) 900 °C in a tube furnace. Figure S4: The OM images of (a–c) 1L and (d–f) 2L WSe_2_ nanosheets grown at 800 and 900 °C with growth times of 1, 5, and 10 min, respectively. Figure S5: The output characteristics of photodetectors based on (a,c) 1L and (b,d) 2L WSe_2_ nanosheets at Vg = 0 V under (a,b) 532 and (c,d) 633 nm lasers with various power densities. Figure S6: The photocurrent and photoresponsivity of (a,c) 1L and (b,d) 2L WSe_2_ devices illuminated by (a,b) 532 and (c,d) 633 nm lasers as a function of laser power density. Table S1: Performance comparison of this work to the previously reported TMDC-based photodetector. Figure S7: The PDR plots of photodetectors based on 1L and 2L WSe_2_ nanosheets under (a) 532 and (b) 633 nm lasers with power densities of 5.7 and 5.4 mW/mm^2^, respectively. Figure S8: The PDR plots of photodetectors based on the 1L and 2L WSe_2_ nanosheets under (a,c) 532 and (b,d) 633 nm lasers, respectively, as a function of the laser power density. Figure S9: Response and recovery time of photodetectors based on (a–c) 1L and (d–f) 2L WSe_2_ nanosheets under (a,d) 405, (b,e) 532, and (c,f) 633 nm lasers, respectively.
