# Supplementary material for: Controllable Growth of Monolayer and Bilayer WSe2 by Liquid-Phase Precursor via Chemical Vapor Deposition for Photodetection
Source: Nanomaterials (Basel). 2024 Dec 16;14(24):2021. doi: 10.3390/nano14242021 (PMC11728604; doi:10.3390/nano14242021)
Supplement: Supplementary file 1 [file nanomaterials-14-02021-s001.zip › nanomaterials-3344838-supplementary.pdf]

nanomaterials

MDPI

Supplementary Materials

Controllable Growth of Monolayer and Bilayer WSe_2 by Liquid-Phase Precursor via Chemical Vapor Deposition for Photodetection

Siyuan Wang, Pinyi Wang, Hailun Tang, Shilong Yu, Huihui Ye, Xinyu Fang, Jing Ding, Yang Yang and Hai Li *

School of Flexible Electronics (Future Technologies) & Institute of Advanced Materials (IAM), Nanjing Tech University (NanjingTech), 30 South Puzhu Road, Nanjing 211816, China
* Correspondence: iamhli@njtech.edu.cn

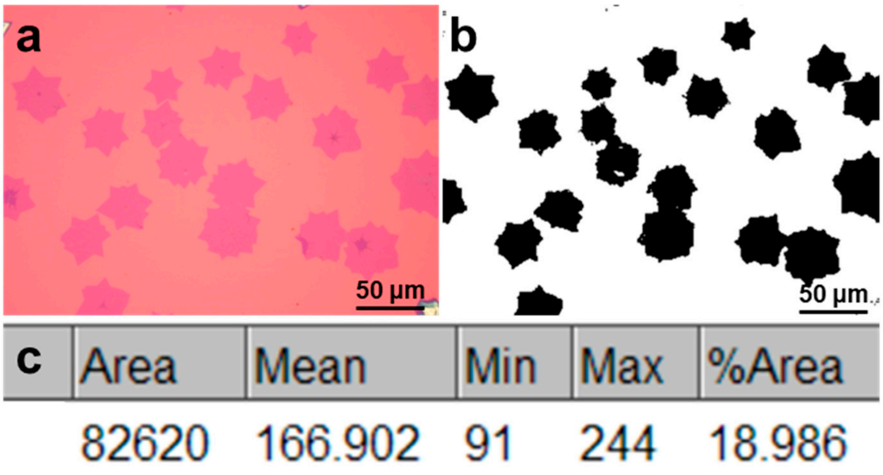

Figure S1. The statistics investigation of the surface area percentage of SiO_2/Si substrate covered by WSe_2 nanosheets. (a) Color image of WSe_2 nanosheets on SiO_2/Si substrate. (b) Gray-scale image of (a) processed by ImageJ. (c) The coverage of WSe_2 nanosheets on SiO_2/Si substrate was statistically analyzed by ImageJ.

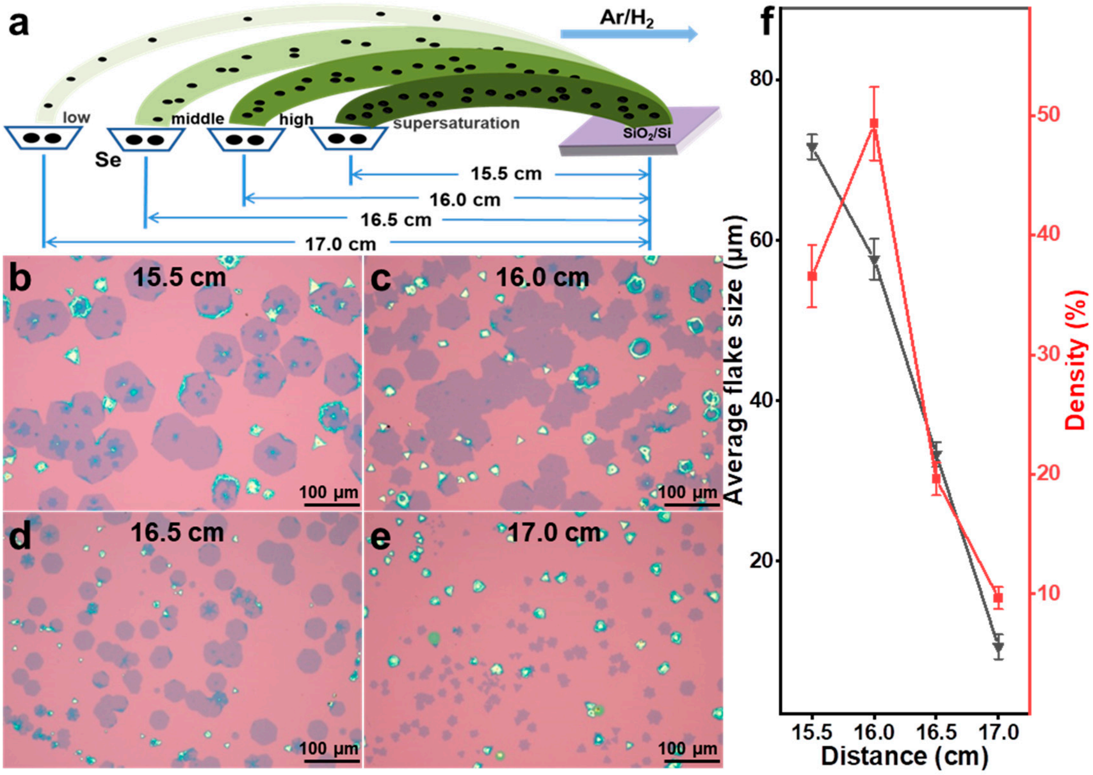

Figure S2. (a) Schematic illustration of the evaporation of Se powder at various distances away from the SiO_2/Si substrate placed at the center of a CVD tube furnace. (b–e) OM images of 1L WSe_2 nanosheets grown at distances between the substrate and selenium powder of (b) 15.5, (c) 16.0, (d) 16.5, and (e) 17.0 cm, respectively. (f) The average flake size and density of 1L WSe_2 nanohseets as a function of distance.

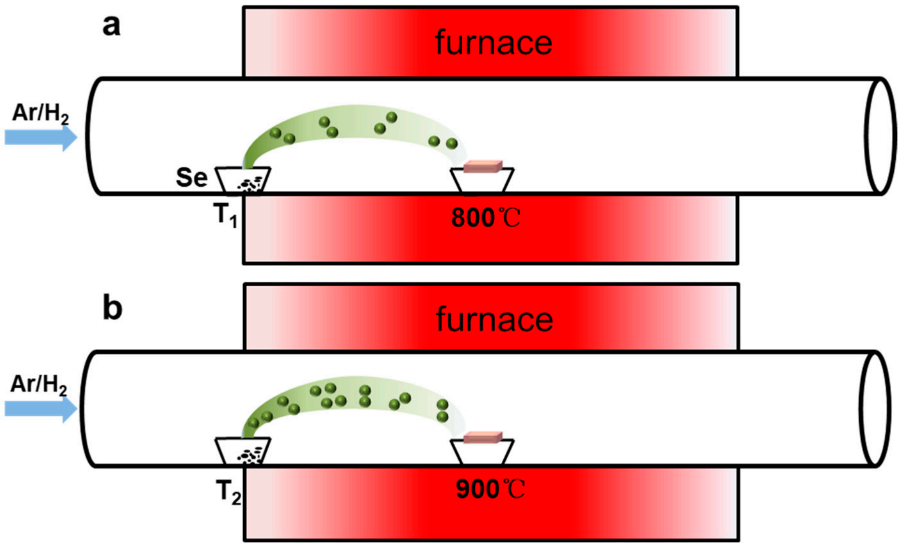

Figure S3. Schematic illustration of the evaporation of Se powder at (a) 800 and (b) 900℃ in a tube furnace.

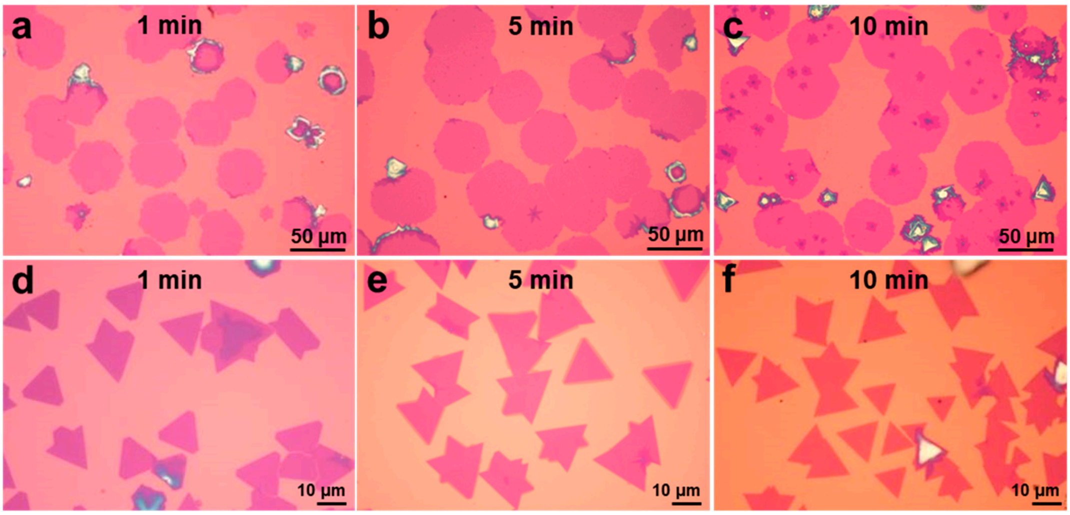

Figure S4. The OM images of (a–c) 1L and (d–f) 2L WSe_2 nanosheets grown at 800 and 900℃ with growth times of 1, 5, and 10 minutes, respectively.

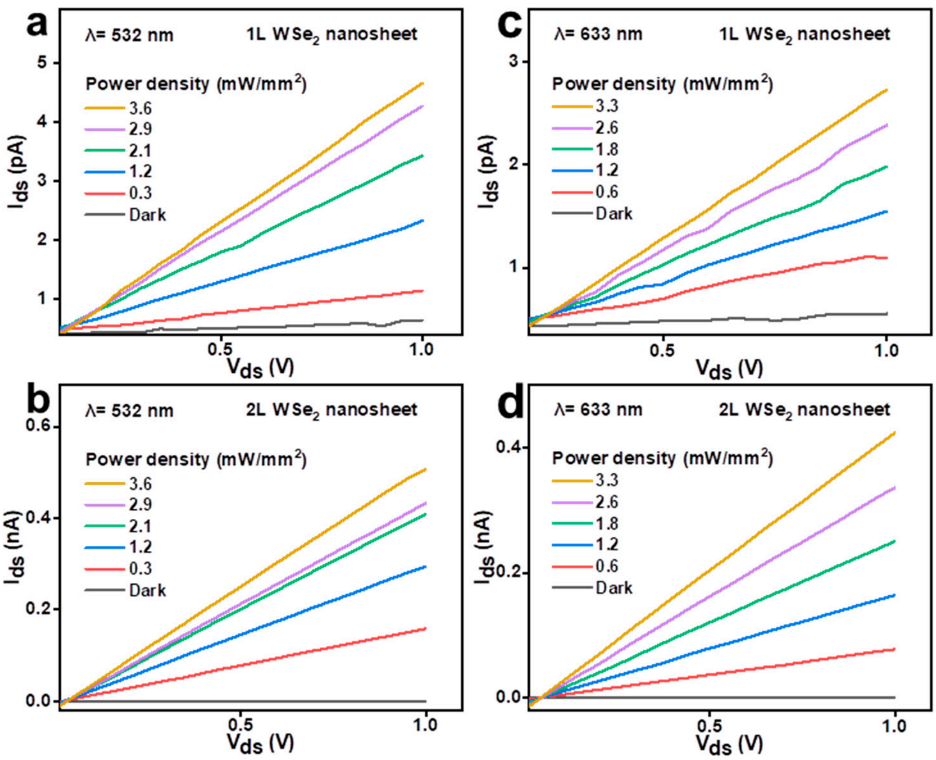

Figure S5. The output characteristics of photodetectors based on (a,c) 1L and (b,d) 2L WSe_2 nanosheets at Vg = 0 V under (a,b) 532 and (c,d) 633 nm lasers with various power densities.

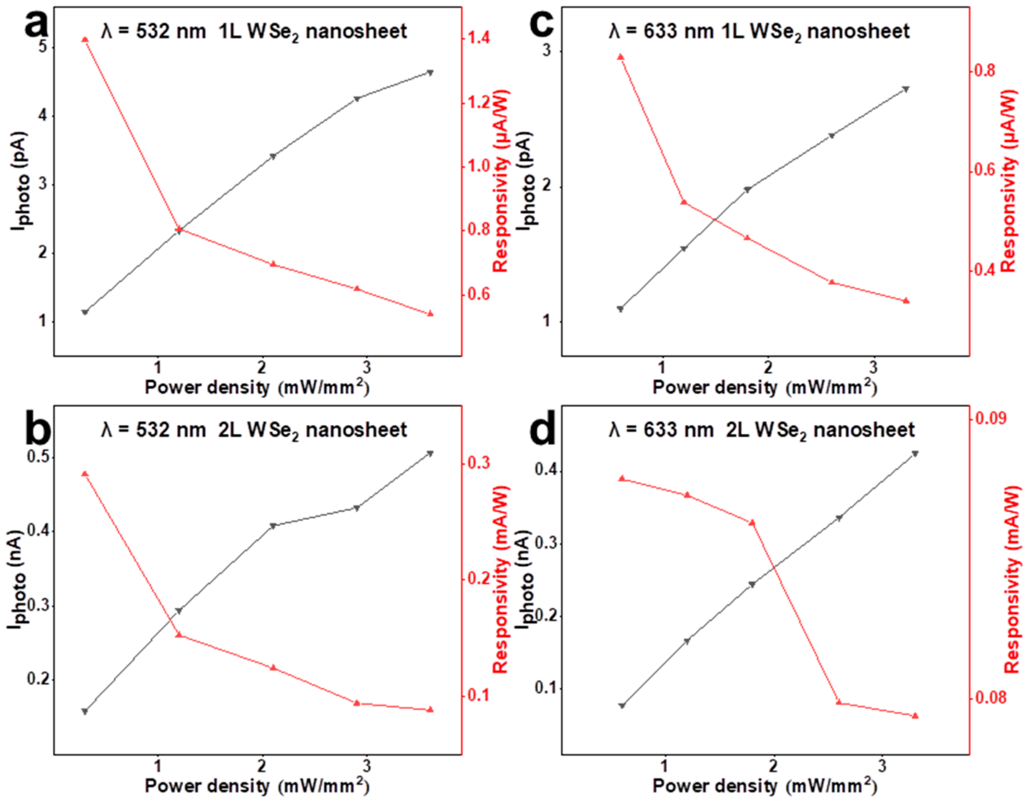

Figure S6. The photocurrent and photoresponsivity of (a,c) 1L and (b,d) 2L WSe_2 devices illuminated by (a,b) 532 and (c,d) 633 nm lasers as a function of laser power density.

Table S1. Performance comparison of this work to the previously reported TMDC-based photodetector.

Material	R (A/W)	D* (cm·$Hz^{1/2}W^{-1}$	EQE (%)	Reference
1L WSe_2	2.44×10^{-6}	1.45×10^{9}	7.46×10^{-4}	This work
2L WSe_2	5.36×10^{-4}	4.43×10^{11}	1.64×10^{-1}	
WSe_2	1.31×10^{-6}	-	-	Nanomaterials 2022, 12, 1854
Te/2D $MoSe_2$	3.28×10^{-1}	8.20×10^{9}	7.90×10^{1}	Adv. Funct. Mater. 2024, 34, 2311134
WSe_2	7.00×10^{-4}	-	1.00×10^{-1}	Nano Lett. 2014, 14, 5846
TiS_3	1.62×10^{8}	4.33×10^{15}	-	Sensors 2023, 23, 4948

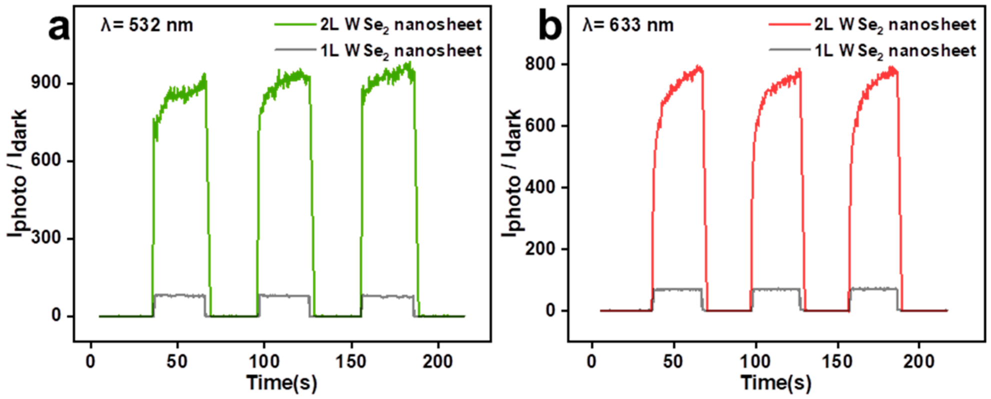

Figure S7. The PDR plots of photodetectors based on 1L and 2L WSe_2 nanosheets under (a) 532 and (b) 633 nm lasers with power densities of 5.7 and 5.4 mW/mm^2, respectively.

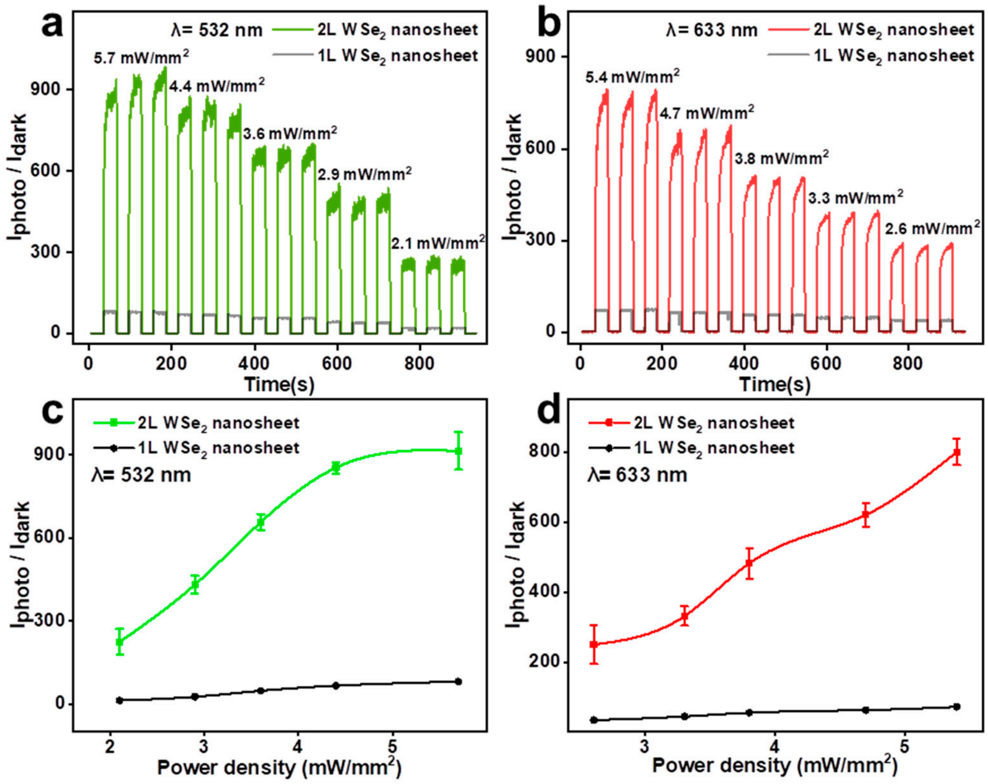

Figure S8. The PDR plots of photodetectors based on the 1L and 2L WSe_2 nanosheets under (a,c) 532 and (b,d) 633 nm lasers, respectively, as a function of the laser power density.

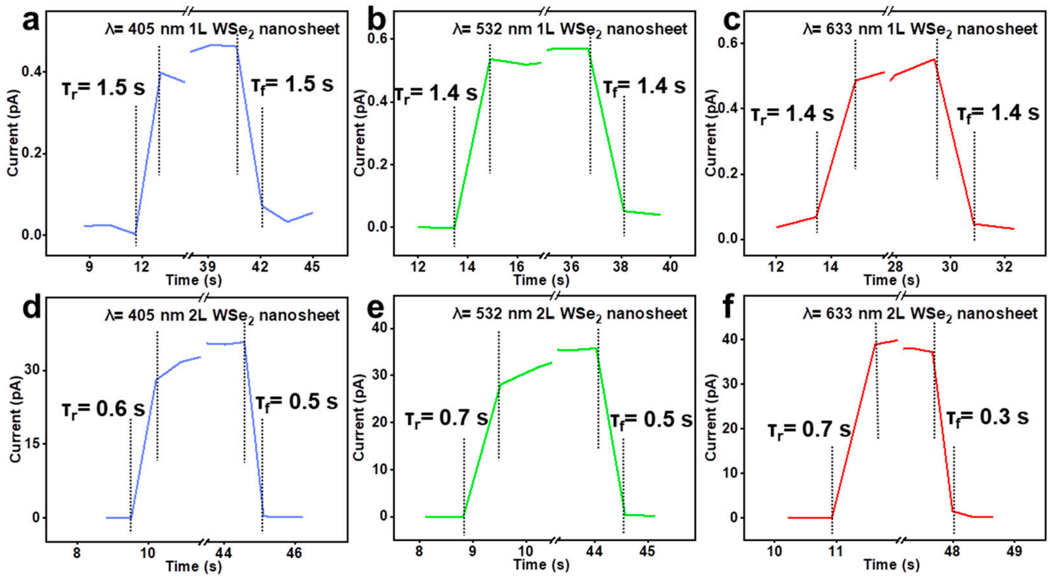

Figure S9. Response and recovery time of photodetectors based on (a–c) 1L and (d–f) 2L WSe_2 nanosheets under (a,d) 405, (b,e) 532, and (c,f) 633 nm lasers, respectively.
